# Genome-wide Association Analysis of Powdery Mildew Resistance in U.S. Winter Wheat

**DOI:** 10.1038/s41598-017-11230-z

**Published:** 2017-09-18

**Authors:** Na Liu, Guihua Bai, Meng Lin, Xiangyang Xu, Wenming Zheng

**Affiliations:** 1 College of Life Science, Henan Agricultural University, Zhengzhou, Henan 450002 China; 2 USDA–ARS Hard Winter Wheat Genetics Research Unit, Manhattan, KS 66506 USA; 3 Department of Agronomy, Kansas State University, Manhattan, KS 66506 USA; 4 Wheat, Peanut and Other Field Crops Research Unit, USDA-ARS, Stillwater, OK 74075 USA

## Abstract

Wheat powdery mildew (PM), caused by *Blumeria graminis* f. sp. *tritici*, is a major fungal disease of wheat worldwide. It can cause considerable yield losses when epidemics occur. Use of genetic resistance is the most effective approach to control the disease. To determine the genomic regions responsible for PM resistance in a set of U.S. winter wheat and identify DNA markers in these regions, we conducted a genome-wide association study on a set of 185 U.S. winter wheat accessions using single nucleotide polymorphism (SNP) markers from 90 K wheat SNP arrays. We identified significant SNP markers linked to nine quantitative trait loci (QTLs) and simple sequence repeats (SSR) markers linked to three QTLs for PM resistance. Most of the QTLs in the US winter wheat population have been reported previously, but some such as these on chromosomes 1A, 6A and 1B have not been reported previously, and are likely new QTLs for PM resistance in U.S. winter wheat. The germplasm with immunity to PM are good sources of resistance for PM resistance breeding and the markers closely linked to the QTLs can be used in marker-assisted selection to improve wheat PM resistance after further validation.

## Introduction

Wheat (*Triticum aestivum* L.) is one of the staple food crops and serves nearly 35% of the world populations^1^. Powdery mildew (PM), caused by *Blumeria graminis* f. sp. *tritici*, is one of the most destructive wheat diseases worldwide^2,3^. PM occurs in wheat fields where high moisture is available^4,5^ and its epidemics can cause a significant grain yield reduction up to 34%^6–8^. As high demand for wheat yield increase to meet world-growing population, PM is becoming more severe because denser plant canopy of modern cultivars under high nitrogen fertilizer supply favors PM development. Use of resistant cultivars is the most economical environmental friendly approach to reduce the yield loss due to the disease^9^. However, rapid development of new races of the pathogen can quickly overcome the host resistance and result in PM outbursts^10^.

Wheat PM resistance is conditioned by both race specific and non-race specific resistance genes. To date, 55 resistant genes have been formally designated (*Pm1*-*Pm55*) and mapped to various chromosomes^11,12^. *Pm1a* was the first PM resistance gene identified on the long arm of chromosome 7A from a Canadian wheat cultivar Axminster^13–17^. Since then, other genes have been identified and tightly linked DNA markers have been reported. Some PM resistance genes, such as *Pm2* on 5DS^13,14^, *Pm3* on 1AS^14,18,19^, *Pm18* on 7A^13^, were identified from bread wheat, whereas others were introgressed into wheat from wheat close relatives, including *Pm4a* on 2AL from *T. monococcum, Pm4b* on 2AL from *T. carthlicum* ^14^, *Pm12* on 6BS from *Aegilops speltoides* ^20^, *Pm13* on 3B and 3D from *Ae. longissima* ^21^, and *Pm25* on 1A from *T. monococcum* ^22^. Some *Pm* genes were identified from non-progenitor species of wheat, including *Pm7*, *Pm8*, *Pm17*, *Pm20* and *PmCn17* from *Secale cereal*, *Pm21* from *Haynaldia villosa*, and *Pm40* and *Pm43* from *Thinopyrum intermedium* ^23–30^. Most of these genes have been used in breeding for PM resistance in wheat. However, new PM pathogen races may easily overcome the resistance conferred by these major genes^31^. Therefore, it is very important to continuously explore new resistance genes to diversify resistance sources against rapidly evolving pathogen races.

Molecular markers have been successfully used to tag genes or quantitative trait loci (QTLs) and to estimate their effects^32–35^. Traditionally, bi-parental mapping populations are used to determine the locations of resistance genes or QTLs in one or two cultivars^36–40^. However, markers linked to a QTL identified from a specific mapping population may not be useful for marker-assisted breeding in other breeding populations^41^. More recently, association mapping (AM) has been used for identification and dissection of disease resistance genes or QTLs in many plant species^42^. Unlike bi-parental QTL mapping, AM does not require development of populations, and can quickly assemble a population by collecting a set of diversity germplasm^43^. Tightly linked markers to QTLs identified from AM study have high potential to be used for selection of the identified genes or QTLs in breeding.

Population structure displayed a systematic difference in allele frequencies between subpopulations^44^. The unequal distribution of alleles among the subpopulations may result in an increase in false association^42,45^. Population structure (Q) and genetic relatedness (Kingship) can be integrated together into a statistical model for association analysis to reduce such false association^46,47^.

In this study, we used the AM approach to study PM resistance in a set of elite breeding lines from U.S. winter wheat breeding programs. The objectives of this study were to 1) determine the genes or QTLs for PM resistance in a panel of US winter wheat germplasm, and 2) identify SNP and SSR markers associated with the QTLs or genes for marker-assisted selection.

## Results

### Powdery mildew resistance in the association mapping population

**Figure 1:**
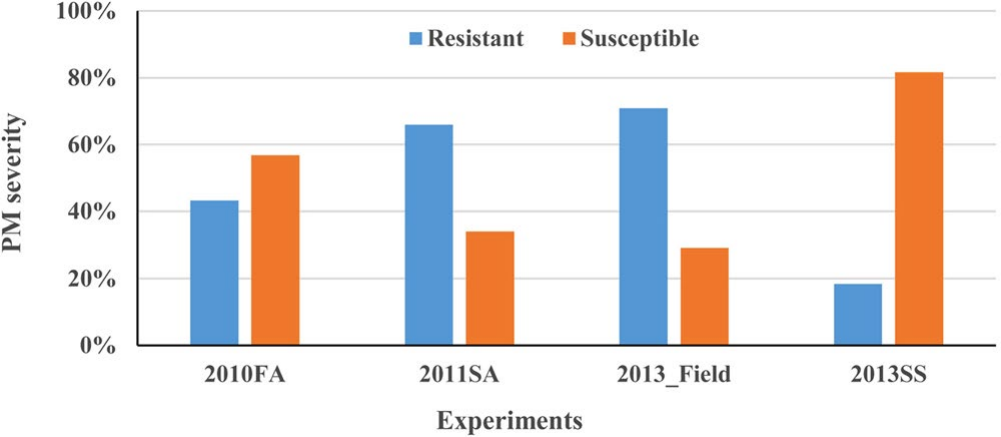
Percentage of powdery mildew resistant and susceptible accessions in the association mapping population evaluated at adult plant stage in fall 2010 (2010FA) and spring 2011 (2011SA) greenhouse experiments and field 2013 experiment (2013_Field), and at seedling stage in spring 2013 greenhouse experiment (2013SS).

The PM scores of the 185 wheat accessions in the association mapping population ranged from 0 to 90%. In seedling stage, about 18% accessions were resistant when <20% severity was classified resistant; whereas in adult plant stage, 43% and 66% of accessions, respectively, were resistant in the two greenhouse experiments and 71% of the tested accessions showed resistance in the field experiment (Supplemental Table S1, Fig. 1). Thirty-three lines including 20 soft wheat lines and 13 hard wheat lines showed immune response in adult plant stage for all three experiments and they are good sources of resistance to PM. About 50 lines showed trace PM at adult stage in one of the three experiments. The results suggest that more tested accessions, especially soft winter wheat, had adult plant resistance than seedling resistance. For adult plant resistance, more resistant accessions were observed in the 2013 field and the 2011 greenhouse experiments than the 2010 greenhouse experiments. The correlation coefficients of PM scores were all significant among the four experiments and was the highest between 2010 and 2011 greenhouse experiments (r = 0.762) and the lowest between 2013 field and 2013 greenhouse (r = 0.466) experiments, suggesting that some adult plant resistance genes expressed in the field experiment might be different from these expressed in seedling stage in the greenhouse experiments.

### Population structure

**Figure 2:**
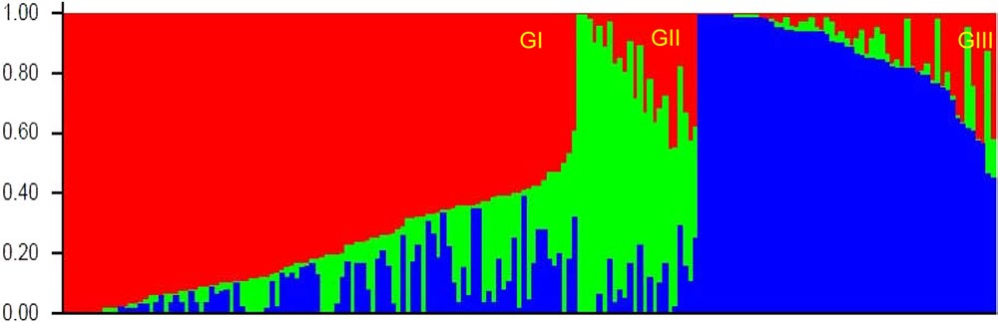
Structure analysis devided the population of 185 U.S. wheat accessions into three groups (GI, GII and GIII).

**Figure 3:**
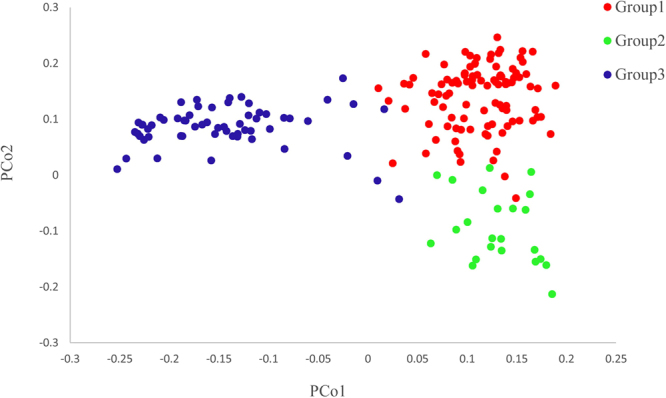
Principal coordinate analysis (PCoA) separated the population of 185 U.S. wheat accessions into three groups that correspond to the three groups derived from structure analysis.

Structure analysis indicated that the population could be divided into three groups (Fig. 2). Group I (102 accessions) and Group II (23 accessions) are mainly hard winter wheat, whereas Group III (60 accessions) is mainly soft winter wheat. One quarter of accessions in the Group I are hard white winter wheat (HWWW) and others are hard red winter wheat (HRWW). Group II consists of all HRWW with most of accessions having Jagger (13 accessions) in their pedigrees. Principal coordinate analysis (PCoA) and a similarity matrix heat-map (Supplementary Fig. S1) confirmed the three groups derived from structure analysis (Fig. 3).

AMOVA showed that individuals within groups accounted for 89% of the genetic variation, whereas only 11% was explained by the variation among the groups (Table 1).

**Table 1:** Analysis of molecular variance on the association mapping population of 185 winter wheat accessions using SNP data.

Sources	df	SS	MS	Est. var.	%	*P* value
Among pops	2	1939.733	969.866	15.826	11%	0.001
Within pops	182	22579.348	124.062	124.062	89%	
Total	184	24519.081		139.889	100%	

### Markers significantly associate with PM resistance

**Figure 4:**
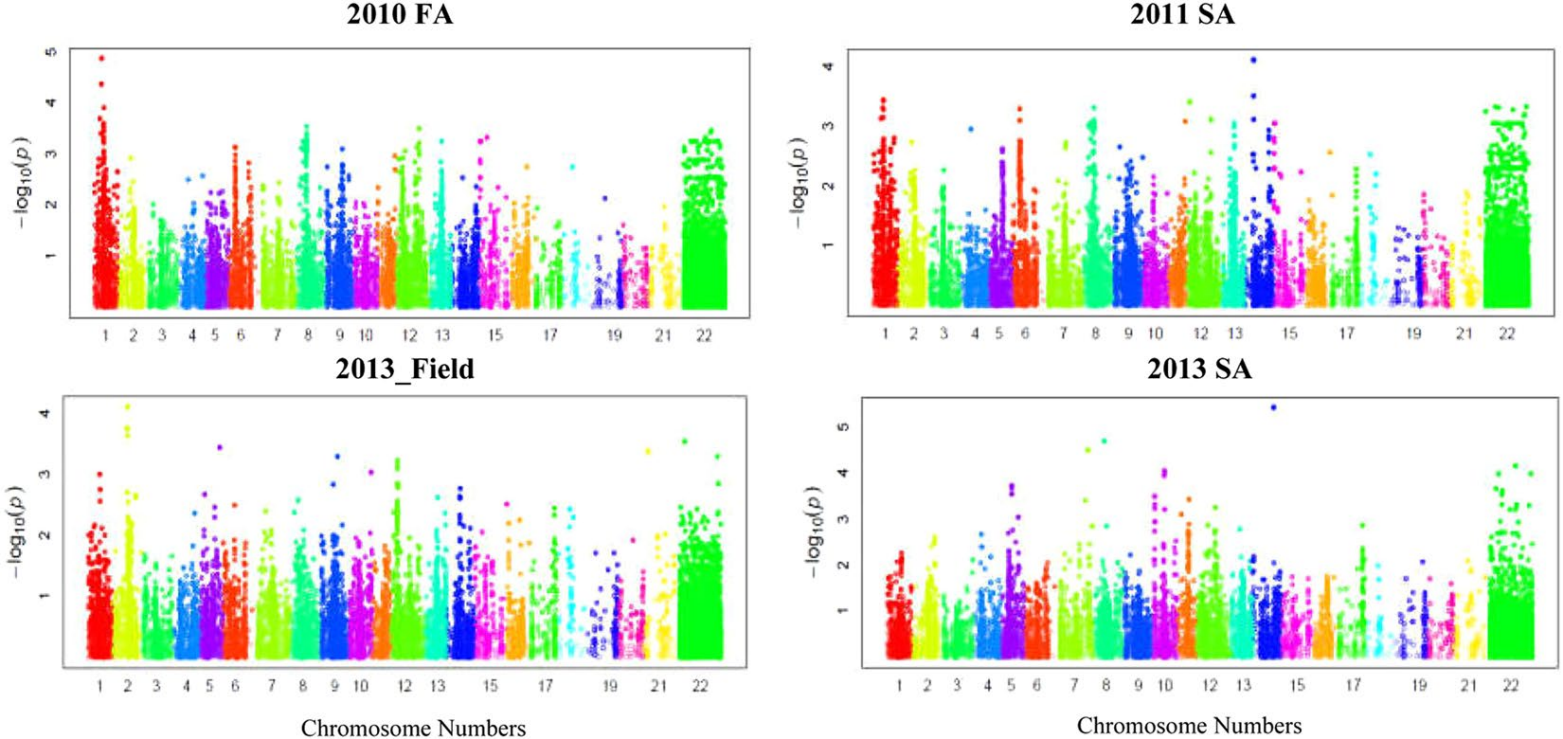
Manhattan plots for SNPs associated with powdery mildew resistance in wheat.

**Figure 5:**
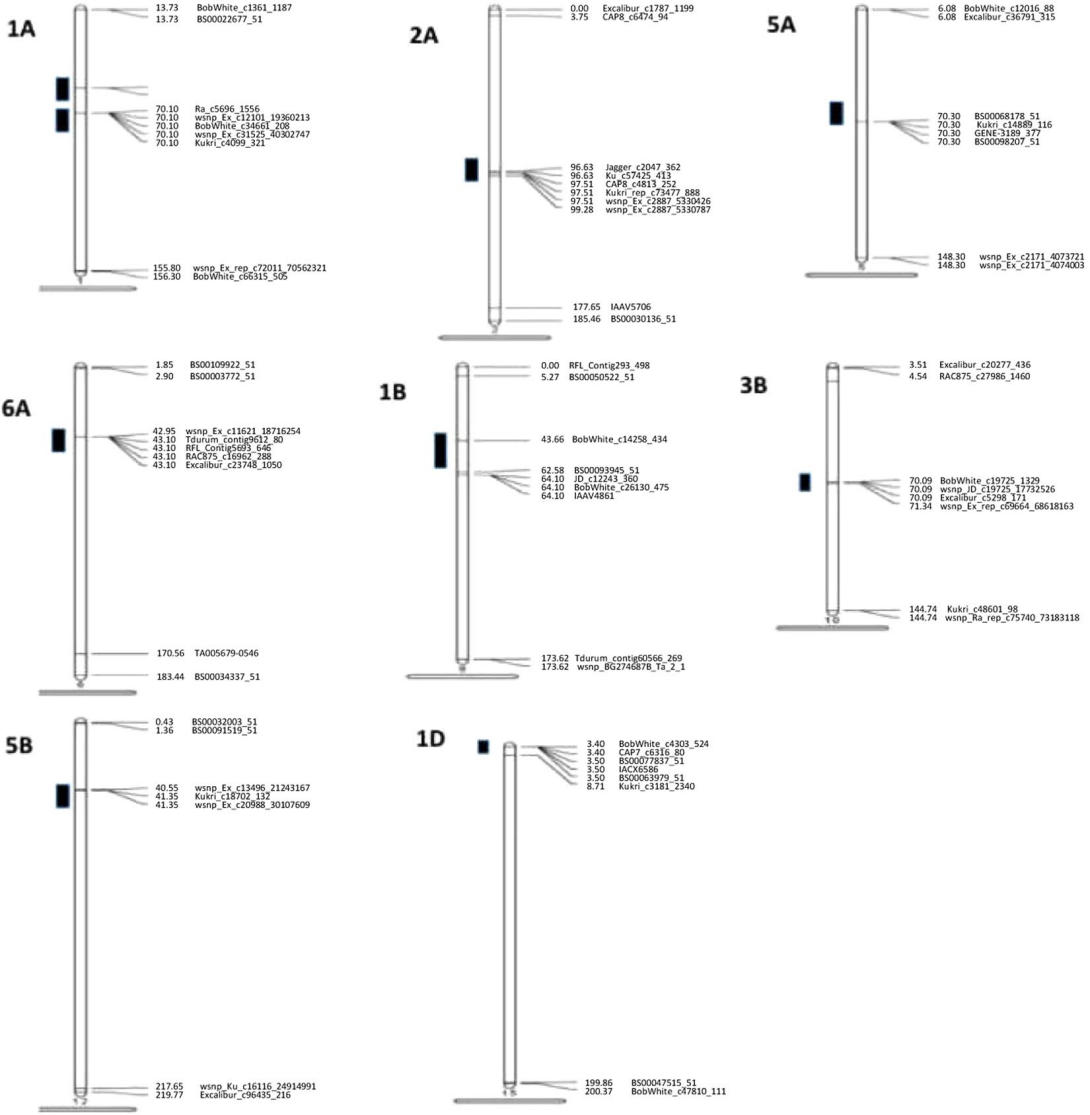
Linkage maps to show chromosomal locations of the significant QTLs for powdery mildew resistance from association mapping using 185 winter wheat accessions.

**Figure 6:**
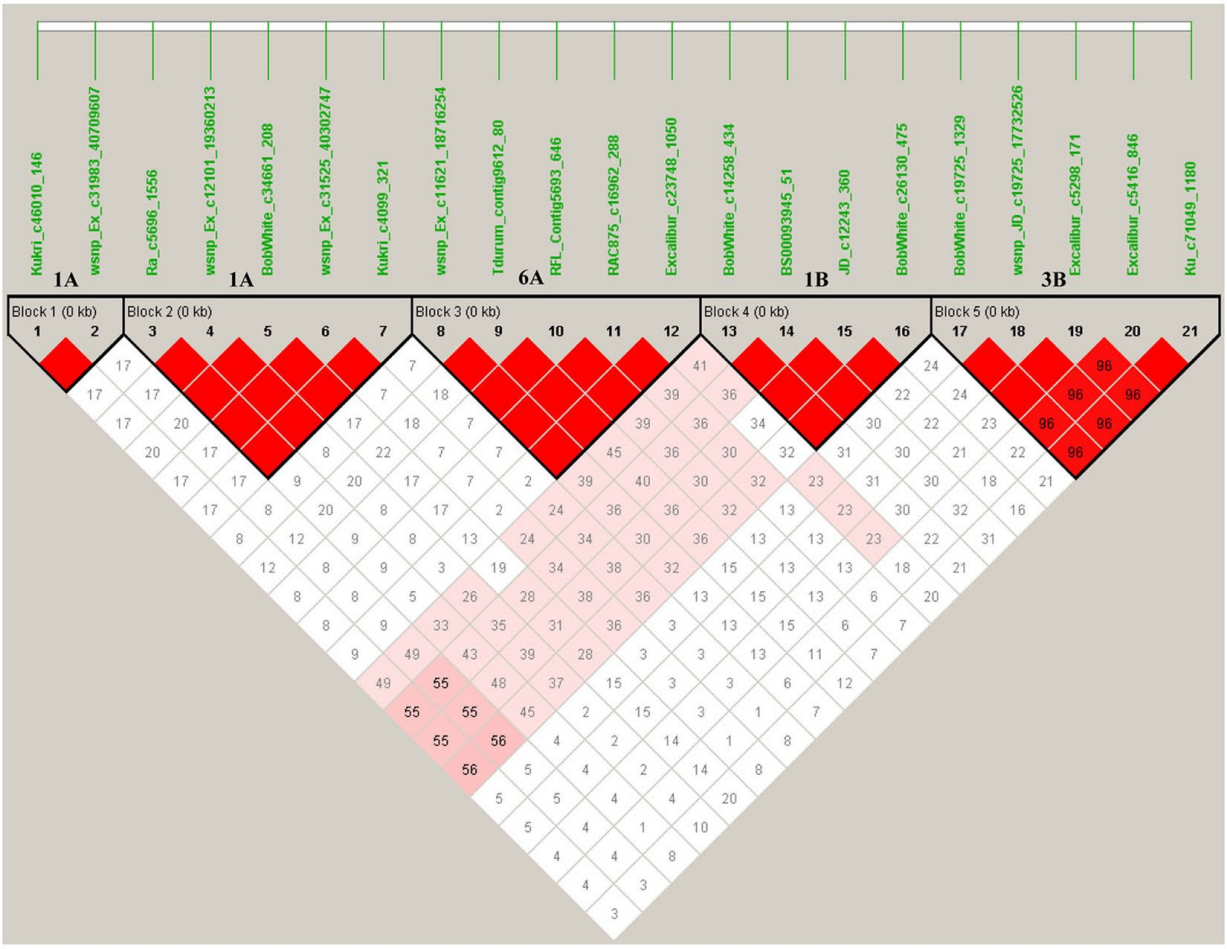
Linkage Disequilibrium (LD) of some significant SNP markers.

The optimal model was selected based on the observed *P*-values and the expected *P*-values for each trait (Q–Q plots). The MLM model including population structure (Q) and kingship (K) showed a better fit than GLM, and thus was selected for further association analysis (Supplementary Fig. S2). A total of 37 SNPs showed significant associations with PM resistance (Table 2, Fig. 4) and were mapped to nine regions on eight chromosomes (Fig. 5). SNPs on three chromosomes (6A, 1B and 1D) were significantly associated with PM resistance in at least two experiments. Other five chromosome regions showed a significant association with PM resistance in only one experiment. On chromosome 6A, three SNPs (wsnp_Ex_c11621_18716254, RFL_Contig5693_646, RAC875_c16962_288) were significant in the 2010 greenhouse experiment and two SNPs (Tdurum_contig9612_80, Excalibur_c23748_1050) were significant in the 2011 greenhouse experiment. The five SNPs were located within 1 cM (Table 2) and mapped in the same LD group (Fig. 6), and thus they are tightly linked markers and most likely associated with the same gene that showed a significant effect on PM resistance in the two experiments. Four SNPs on chromosome 1B were significant in the 2010 and 2011 greenhouse experiments. One of them was mapped in the chromosome position 43.66 cM and other three were mapped in about 19 cM away on the reference map (Table 2, Fig. 5)^48^. LD analysis indicated they were in the same LD (Fig. 6) and likely the same QTL for PM resistance. Three SNPs that were mapped within a 5 cM region on the chromosome 1D were also significantly associated with PM resistance in the 2010 and 2011 greenhouse experiments (Table 2), and they are more likely linked to the same QTL on the chromosome 1D.

**Table 2:** Significant SNP markers associated with wheat powdery mildew resistance evaluated in the greenhouse experiments of fall 2010 (2010FA) and spring 2011 (2011SA) and the field experiment of 2013 (2013_Field) for adult plant resistance, spring 2013 greenhouse for seedling resistance (2013SS).

Marker name	Chromosome	Position^*^ (cM)	2010FA		2011SA		2013_Field		2013SS		Resistance allele	Sensitive allele
			*P* value	*R* Squared	*P* value	*R* Squared	*P* value	*R* Squared	*P* value	*R* Squared		
Kukri_c46010_146	1A	56.39	4.31E-05	0.09	—	—	—	—	—	—	C	T
wsnp_Ex_c31983_40709607	1A	56.39	4.31E-05	0.09	—	—	—	—	—	—	C	T
Ra_c5696_1556	1A	70.1	2.85E-04	0.07	—	—	—	—	—	—	T	C
wsnp_Ex_c12101_19360213	1A	70.1	3.71E-04	0.06	—	—	—	—	—	—	T	C
BobWhite_c34661_208	1A	70.1	4.81E-04	0.06	—	—	—	—	—	—	G	T
wsnp_Ex_c31525_40302747	1A	70.1	5.90E-04	0.06	—	—	—	—	—	—	T	C
Kukri_c4099_321	1A	70.1	5.90E-04	0.06	—	—	—	—	—	—	C	T
Jagger_c2047_362	2A	96.63	—	—	—	—	1.76E-04	0.07	—	—	G	A
Ku_c57425_413	2A	96.63	—	—	—	—	1.76E-04	0.07	—	—	G	A
CAP8_c4813_252	2A	97.51	—	—	—	—	7.72E-05	0.07	—	—	A	C
Kukri_rep_c73477_888	2A	97.51	—	—	—	–	1.81E-04	0.06	—	—	T	C
wsnp_Ex_c2887_5330426	2A	97.51	—	—	—	—	1.81E-04	0.06	—	—	T	C
wsnp_Ex_c2887_5330787	2A	99.28	—	—	—	—	2.29E-04	0.06	—	—	T	C
BS00068178_51	5A	70.3	—	—	—	—	—	—	1.91E-04	0.08	T	C
Kukri_c14889_116	5A	70.3	—	—	—	—	—	—	1.91E-04	0.08	T	C
GENE-3189_377	5A	70.3	—	—	—	—	—	—	2.73E-04	0.07	C	T
BS00098207_51	5A	70.3	—	—	—	—	—	—	2.73E-04	0.07	T	C
wsnp_Ex_c11621_18716254	6A	42.95	7.32E-04	0.06	—	—	—	—	—	—	T	C
Tdurum_contig9612_80	6A	43.1	—	—	5.12E-04	0.05	—	—	—	—	T	C
RFL_Contig5693_646	6A	43.1	7.32E-04	0.06	—	—	—	—	—	—	A	G
RAC875_c16962_288	6A	43.1	7.32E-04	0.06	—	—	—	—	—	—	C	T
Excalibur_c23748_1050	6A	43.1	—	—	7.92E-04	0.05	—	—	—	—	A	G
BobWhite_c14258_434	1B	43.66	5.67E-04	0.06	9.12E-04	0.05	—	—	—	—	A	C
BS00093945_51	1B	62.58	5.39E-04	0.06	—	—	—	—	—	—	A	G
JD_c12243_360	1B	64.1	4.47E-04	0.06	8.45E-04	0.05	—	—	—	—	C	T
BobWhite_c26130_475	1B	64.1	5.67E-04	0.06	9.12E-04	0.05	—	—	—	—	C	T
BobWhite_c19725_1329	3B	70.09	—	—	—	—	—	—	8.87E-05	0.09	G	A
wsnp_JD_c19725_17732526	3B	70.09	—	—	—	—	—	—	9.96E-05	0.09	A	G
Excalibur_c5298_171	3B	70.09	—	—	—	—	—	—	1.14E-04	0.08	C	T
Excalibur_c5416_846	22	-	—	—	—	—	—	—	6.18E-04	0.07	A	T
Ku_c71049_1180	22	-	—	—	—	—	—	—	6.13E-04	0.07	T	C
wsnp_Ex_c13496_21243167	5B	40.55	—	—	—	—	7.03E-04	0.05	—	—	A	G
Kukri_c18702_132	5B	41.35	—	—	—	—	5.89E-04	0.05	—	—	A	G
wsnp_Ex_c20988_30107609	5B	41.35	—	—	—	—	8.10E-04	0.05	—	—	G	A
BS00077837_51	1D	3.5	5.67E-04	0.06	9.12E-04	0.05	—	—	—	—	A	G
IACX6586	1D	3.5	5.67E-04	0.06	9.12E-04	0.05	—	—	—	—	T	C
Kukri_c3181_2340	1D	8.71	5.67E-04	0.06	9.12E-04	0.05	—	—	—	—	T	G

Note: “—” denotes not significant. ^*^The position of markers on wheat consensus SNP map.

In five other chromosome regions that were significantly associated with PM resistance in a single experiment (Table 2), two SNPs, Kukri_c46010_146 and wsnp_Ex_c31983_40709607, which were mapped at 56.39 cM on chromosome 1A (Table 2), were significant in the 2010 greenhouse experiment. Five other significant SNPs (Ra_c5696_1556, wsnp_Ex_c12101_19360213, BobWhite_c34661_208, wsnp_Ex_c31525_40302747, and Kukri_c4099_321) were mapped at 14 cM away on the same chromosome (Table 2). The two sets of markers showed no LD and more likely associated with two different QTLs for PM resistance (Fig. 6). Six SNPs were mapped within a 3 cM region on the chromosome 2A and all significant in 2013 field experiment (*R* ^2^ = 0.073, *P* < 0.000077), suggesting one PM resistance QTL is more likely in the region. Three SNPs mapped within a 1 cM region on 5B were also significant in 2013 field experiment (*R* ^2^ = 0.078, *P* < 0.0003). Four SNPs on chromosome 5A (*P* < 0.0003) and five SNPs on chromosome 3B (*P* < 0.0007) were significant in the 2013 greenhouse experiment. The four SNPs on 5A were mapped together, suggesting that one QTL for seedling resistance may link to these SNPs (Table 2). SNPs on 3B either were mapped together or shared the same LD, suggesting they more likely link to a single gene for seedling resistance on the chromosome 3B (Table 2, Fig. 6).

Among 457 SSR markers from all 21 chromosomes screened, three were significant for PM resistance. Xscm0009 on chromosome 1A was significantly associated with PM resistance in 2010 greenhouse experiments (*R* ^2^ = 0.08, *P* < 0.0007, Table 3). Two other markers, Xcfd9-2 on 3D and Xcfd95 on 6D, were significant in the 2013 field experiment.

**Table 3:** Significant SSR markers associated with wheat powdery mildew resistance evaluated in the greenhouse experiments of fall 2010 (2010FA) and spring 2011 (2011SA) and the field experiment of 2013 (2013_Field) for adult plant resistance, spring 2013 greenhouse for seedling resistance (2013SS).

Test Methods	Marker name	Chromosome	2010FA		2011SA		2013_Field		2013SS	
			*P* value	*R* Squared	*P* value	*R* Squared	*P* value	*R* Squared	*P* value	*R* Squared
MLM	Xscm0009	1 A	7.09E-04	0.08	—	—	—	—	—	—
	Xcfd9-2	3D	—	—	—	—	5.92E-04	0.09	—	—
	Xcfd95	6D	—	–	—	—	3.78E-04	0.07	—	—

Note: “–” denotes not significant.

The adult plant PM resistance level of each line is highly correlated with number of QTLs in the lines (r = 0.6083). These QTLs showed obvious additive effects. When a line carried four or more QTLs, it usually showed immune response to PM infection.

## Discussion

Population structure may result in false associations between traits and markers if it is not properly treated during analysis^49^. In this study, both structure analysis and PCoA stratified the AM panel of 185 accessions into three groups and AMOVA also indicated significant population differentiation (*P* < 0.001), demonstrating the presence of obvious structure in the population. Wheat cultivars from NRPN were mostly clustered to Group I and wheat cultivars from RGON and SRPN were mostly clustered to Group I and Group II, whereas accessions in USSRWWN and UESRWWN showed higher PM resistance and clustered into Group III. The result indicated that PM resistance was partly influenced by the geographic distribution.

A significantly higher level of variation was detected within a population (89%) than among populations (11%) by AMOVA, indicating that a high level of genetic diversity, but not genetic divergence, was observed in US winter wheat, therefore, breeding selection has played a significant role in maintaining genetic diversity in the breeding populations.

Using the AM panel in this study, we identified SNP markers linked to nine QTLs for PM resistance and SSR markers linked to three QTLs for PM resistance on the chromosomes 1A, 2A, 5A, 6A, 1B, 3B, 5B, 1D, 3D and 6D. Among them, SNPs closely linked to the three genes on the chromosomes 6A, 1B, 1D were significant in adult plant stage in two experiments, suggesting these genes may have stable adult plant resistance to multiple races presented in both environments.

When significant QTLs identified in the current study were compared with the QTLs reported previously, we found that several QTLs have been mapped to the similar positions where PM resistance genes were reported previously. The seven SNPs that were significantly linked to PM resistance QTL in the 2010 field experiment were mapped to two locations of the chromosome 1A at 15 cM apart, and LD analysis suggested that they were linked to two different genes. *Pm3* has been previously reported on the chromosome 1A^50–54^; *Pm 17* on chromosome 1RS.1AL was mapped about 1.5 cM away from the IAG95-CA (RFLP)/CT355 (AFLP) markers^55^; and *Pm25* on chromosome 1A was mapped at about 12.8 cM from a RAPD marker OPA04950^22^. Five SNP-associated QTLs on the chromosome 1A are more than 40 cM away from *Pm3*, therefore neither of them are *Pm3*. However, this QTL could be the same or a closely linked gene to *QPm.caas-1A* between SSR marker Xbarc148 and Xwmc550^56^ according to the marker sequence position in the W7984 reference map (Supplemental Table S2). The SSR marker, Xscm0009 was associated with PM resistance in the greenhouse experiments of 2010. The banding pattern of Xscm0009 showed that the marker is in 1A/1 R translocation, which suggests the linked gene is most likely *Pm17*. For other two SNPs on chromosome 1A, known linked genes can not be found based on available information, and they may link to a novel gene. However further research is needed to determine its identity.

*Pm4a*, *Pm4b*, *Pm4c* (or *Pm23*), *Pm4d*, *PmDR147*, *PmPS5A* and *PmLK906* were identified on the chromosome 2A in previous reports^14,57–60,61,62^. Among them, *Pm4a* was about 1.5 cM away from the marker Xbcd292^14^ and *QPm.inra-2A* is linked to the SSR marker *Xgwm275* that is also in the vicinity of *Pm4a* ^63^. In this study, the QTL on 2 A was mapped to a chromosome region near *QPm.inra-2A* according to the marker sequence position in the W7984 reference map (Supplemental Table S2) therefore it may be the same QTL as *QPm.inra-2A*.

*Pm2026* was mapped to the distal portion of chromosome 5 AL and flanked by SSR markers Xcfd39 and Xgwm126^64^. The QTLs *QPm.sfr-5A.1*, *QPm.ttu-5A*, *QPm.sfr-5A.2*, *QPm.sfr-5A.3* and *QPm.nuls-5A* were also reported on the chromosome 5A^65–67^. The QTLs *QPm.sfr-5A.1*, *QPm.sfr-5A.2*, *QPm.sfr-5A.3* were linked to RFLP markers^66^. *QPm.ttu-5A* and *QPm.nuls-5A* were mapped in the marker intervals Xgwm186-Xgwm415 and Xgwm617b-Xwmc327, respectively^65,67^. In the current study, the 5A QTL is likely different from the QTLs *QPm.ttu-5A*, *QPm.nuls-5A* and *Pm2026* based on its marker position in the W7984 reference (Supplemental Table S2). Whether the 5A QTL is the one of the previously reported genes remains to be determined due to lack of common markers among the QTLs reported in different studies.

One QTL was identified on chromosome 3B in this study. This QTL is likely *PmHNK*, a gene was previously mapped at 3.8 cM away from the SSR marker Xwmc291^68^ according to the W7984 reference. The map locations of SNPs, Excalibur_c5416_846 and Ku_c71049_1180, are not available, but a strong LD between the two SNPs and the markers mapped on the chromosome 3B located the two SNPs on the same chromosome (Fig. 6).

*Pm30, Pm36, PmAS846* and *MIVE29* were all mapped on chromosome 5B in previous studies. Liu *et al*.^69^ mapped *Pm30* on the chromosome 5B about 6 cM from the SSR marker Xgwm159/460. Three significant SNPs (wsnp_Ex_c13496_21243167, Kukri_c18702_132, wsnp_Ex_c20988_30107609) mapped on the chromosome 5B in the current study are close to *Pm30* based on the W7984 reference map (Supplemental Table S2), therefore, they are likely the same gene.

*Pm10*, *Pm22*, *Pm24a* and *Pm24b* were previously reported on chromosomes 1D^37,70–72^. Tightly linked markers were identified for *Pm24a* ^37^ and *Pm24b* ^72^. Other QTLs have also been reported in this chromosome including *QPm.inra-1D.1* and *QPm.sfr-1D* ^63,66^. The markers flanking *Pm24a* were Xgwm789/Xgwm603 and Xbarc229 at 2.4 and 3.6 cM to the gene, respectively. *Pm24b* was flanked by Xgwm337 and Xbarc229 at the genetic distances of 3.7 and 1.0 cM, respectively. Here, we identified three significant SNPs linked to a gene on chromosome 1D (Table 2, Fig. 5), which is 2.2 cM away from SSR marker Xbarc229 based on W7984 reference map, and thus the QTL identified in this study is more likely *Pm24*.

Among these nine PM resistance QTLs/genes identified by SNP markers in our study, some are located in the chromosome locations where PM resistance genes have not been reported before, and thus they are likely novel genes for PM resistance (Table 2). Resistance genes *Pm21, Pm31, PmY39-2* and *MIRE* were mapped on chromosome 6A^73–76^. However, the QTL on 6A identified in the current study does not link to any of the genes and is more likely a new QTL for PM resistance.

*Pm8*, *Pm32* and *Pm39* were previously reported on chromosomes 1B^28,67,77^. However, *Pm8* and *Pm32* have not been mapped to date. *Pm39* ^67^ was closely linked to SSR marker Xwmc719, but it is about 56.9 cM away from the 1B QTL identified in this study (Table 2, Fig. 5) according to the W7984 reference map. Several other QTLs have been reported in 1B including *QPm.sfr-1B*, *QPm.ttu-1B*, *QPm.vt-1B*, *QPm.vt-1BL*, *QPm.vt-1B*, *QPm.osu-1B* ^65,66,78–80^, but we can not determine the relationship between the newly identified QTL from this study and previous reported ones due to lack of common markers between these studies.

The SSR markers, Xcfd9-2 on chromosome 3D and Xcfd95 on chromosome 6D, also showed significant associations with PM resistance. *Pm45* was mapped on chromosome 6D flanked by Xcfd80, Xmag6139 and Xmag6140^81^. The QTL detected in this study can be mapped near *Pm45* based on the W7984 reference sequence. The QTL detected on 3D is close to *QPm.inra-3D* that was flanked by Xcfd152 and Xgwm707^63^, hence, they may be the same QTL.

In this study, soft wheat carries at least two resistance QTLs that were identified by SNP markers, and showed a higher level of resistance than hard wheat (Supplemental Table 1). For adult plant, a high level correlation between number of QTLs and PM resistance was observed (r = 0.6083). The PM resistance QTLs showed obvious additive effects. The result indicated that four or more QTLs together produce immune response in adult plant. Therefore, pyramiding four or more of the QTLs using marker-assisted selection can obtain PM immune wheat cultivars.

Our study demonstrates that most of US winter wheat germplasm, especially soft winter wheat, have high levels of PM resistance and carry several QTLs for adult plant resistance to PM. Association mapping effectively identified these genomic regions associated with PM resistance and associated markers linked to these QTLs. They showed obvious additive effect on adult plant PM resistance. The accessions carrying multiple resistance QTLs could be excellent sources of PM resistance for US winter wheat breeding because they are either elite breeding lines or locally adapted cultivars. Future work will be to develop bi-parental populations of the accessions to validate the resistance loci identified in this study and develop user-friendly markers that can be used to accelerate the incorporation of these resistance QTLs into new cultivars.

## Materials and Methods

### Plant materials

A set of 185 elite breeding lines and cultivars were selected from the 2008 U.S Southern (SRPN) and Northern (NRPN) Hard Winter Wheat Regional Performance Nurseries, Regional Germplasm Observation Nurseries (RGON), U.S. Uniform Eastern Soft Red Winter Wheat Nursery (UESRWWN), Uniform Southern Soft Red Winter Wheat Nursery (USSRWWN), and elite breeding lines from Oklahoma State University by removal of sibling lines (Supplemental Table S1). Among these accessions, 130 are hard winter wheat and 55 are soft winter wheat.

### DNA extraction and marker analysis

Leaf tissue at the two-leaf stage was collected into 1.1-ml deep-well plates and dried for 2 d in a freeze-dryer (Thermo Fisher Scientific Inc., Waltham, MA, USA) for DNA isolation. The plates containing dried tissue and a 3.2-mm stainless steel bead in each well were shaken at 25 times per sec for 5 min in a Mixer Mill (Retsch GmbH, Germany). Genomic DNA was extracted using the cetyl trimethyl ammonium bromide method and SSR markers were analyzed using an M13-tailed primer as described by Li *et al*.^82^. All PCR products were separated on an ABI PRISM 3730 DNA Analyzer (Applied Biosystems, Foster City, CA, USA), and marker data were scored using GeneMarker version 1.6 (Soft Genetics LLC, State College, PA, USA) and manually checked twice for accuracy.

Wheat accessions were genotyped using the wheat 90 K SNP assay developed by Illumina Inc. (San Diego, CA, USA). The assay was designed under the protocols of the International Wheat SNP Consortium^83^ and conducted at the USDA Small Grains Genotyping Laboratory in Fargo, ND. SNP genotypes were called using GenomeStudio v2011.1 software (Illumina Inc.). SNPs with minor allele frequency less than 5% or with missing data more than 15% were removed. A total of 21,600 SNPs were scored and used for association analysis.

### Disease evaluation

PM was evaluated in both greenhouse and field experiments at Kansas State University, Manhattan, KS from 2010 to 2013. In the greenhouse experiments, five plants were transplanted into 12.5 × 12.5 cm Tara pots (Hummert International, Topeka, KS) after seven weeks of vernalization at 6 °C. The greenhouse temperatures were set at 22 ± 5 °C during a day with supplemental light of 12 h, and at 17 ± 2 °C during a night. The cultivar, Wesley, was used as the susceptible control in all trials. Field experiments were carried out in Rocky Ford Wheat Disease Nursery in Manhattan, KS. Thirty seeds per accession were sown in a 1.3-m row using a randomized complete block design with two replicates. Naturally occurred *B. graminis* f. sp. *tritici* was used as inoculum for both field and greenhouse experiments.

PM severity was visually estimated as overall percentage of infected leaf area two weeks after anthesis when disease reached maximum levels following Chen and Xu^10^. In each experiment, two replications were evaluated for each accession and a mean value of two replications was used for association analysis. Resistance classification followed Liu *et al*. ^31^, with some modification. In brief, plants without PM symptom (0) or low PM coverage (≤20%) were rated as resistant, whereas plants with >20% PM coverage were rated as susceptible.

### Association analysis

The population structure (Q matrix) was analyzed using the program STRUCTURE version 2.2^84^, with a burn-in length of 10,000 and a total of 10,000 Markov chain Monte Carlo iterations for each *k*. Ten independent runs were carried out for each *k* value. The maximum likelihood of each *k* value, the variance among 10 runs, and the pedigree information of each line were weighted to determine the optimal number of groups. The relative kinship (K) matrix was calculated using SPAGeDi^85,86^. Components of genetic variances among and within groups were estimated by analysis of molecular variance (AMOVA) with 1000 permutations using GenAlEx 6.501^87^.

Association analysis was carried out using both generalized linear model (GLM) and mixed linear model (MLM). GLM includes Q matrix for fixed effects, whereas MLM includes both a Q matrix for fixed effects and a kinship matrix for random effects. The observed *P* values and expected *P* values for each trait (Q-Q plot) were used for model comparison to select the best model^88–90^. Association analysis between SSR and PM severity was conducted using TASSEL 2.1^47,91–93^. Associations between SNP markers and PM severity were determined using the Genome Association and Prediction Integrated Tool (GAPIT)^94^, an R package for genome wide association study (GWAS) and genome prediction (http://www.r-project.org). A threshold of *p* < 0.001 was set up to claim significant associations between SSR or SNP markers and the traits. The genetic positions (cM) of SNP markers on chromosomes were determined based on the 2015 wheat consensus map^48^. The marker-trait associations were cross-referenced against all reported QTLs in the literature and the GrainGenes database (https://wheat.pw.usda.gov/GG3/)^95^. Sequences that harbored significant SNPs were further blasted against the W7984 reference sequences to estimate their putative chromosome positions.

To estimate linkage between significant SNPs, Haploview 4.2 (http://www.broad.mit.edu/mpg/haploview/) was used to calculate the linkage disequilibrium (LD) among all significant markers^96^. Markers in close vicinity with strong LDs were considered to represent the same gene.

## Electronic supplementary material

Supplementary info

Supplementary table S1
