# Supplementary material for: Genome-wide Association Analysis of Powdery Mildew Resistance in U.S. Winter Wheat
Source: Sci Rep. 2017 Sep 18;7:11743. doi: 10.1038/s41598-017-11230-z (PMC5603590; doi:10.1038/s41598-017-11230-z)
Supplement: Supplementary file 1 — Supplementary info [file 41598_2017_11230_MOESM1_ESM.pdf]

SUPPLEMENTARY INFORMATION

Manuscript title

Genome-wide Association Analysis of Powdery Mildew Resistance in U.S. Winter Wheat

Author list:

Na Liu[1,3*], Guihua Bai[2], Meng Lin[3], Xiangyang Xu[4], Wenming Zheng[1]

[1]College of Life Science, Henan Agricultural University, Zhengzhou, Henan, 450002, China.

[2]USDA–ARS Hard Winter Wheat Genetics Research Unit, Manhattan, KS, 66506, USA

[3]Department of Agronomy, Kansas State University, Manhattan, KS, 66506, USA

[4]Wheat, Peanut and Other Field Crops Research Unit, USDA-ARS, Stillwater, OK 74075, USA

*Corresponding author: Na Liu (email: naliu@henau.edu.cn).

Content

Supplementary Figure S1. ....…….…………………………………….………………1

Supplementary Figure S2. ....…….…………………………………….………………2

Supplementary Table S2. .....……………………………………….……………...……3

Supplementary Figure S1 Heatmap of Gowers similarity coefficient to show three distinct clusters among the 185 U.S. wheat accessions.

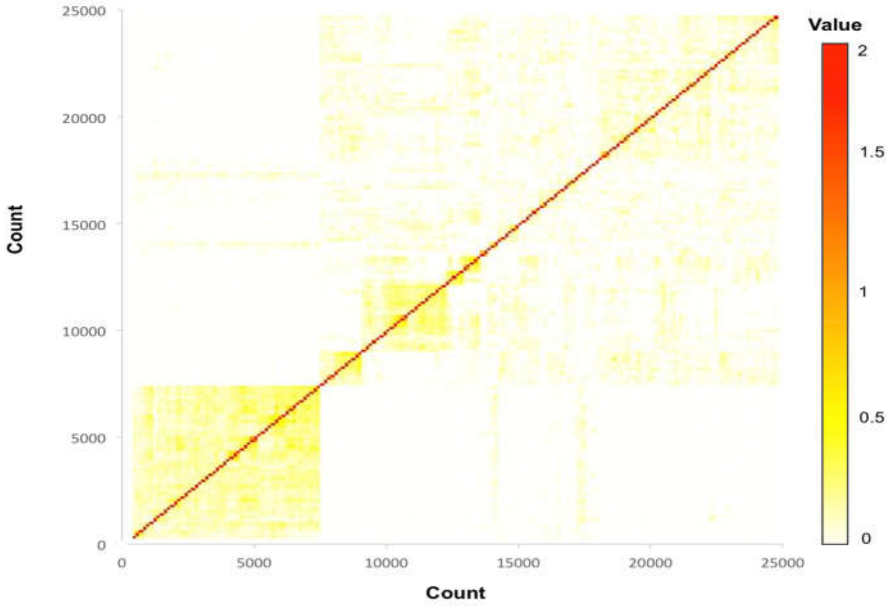

Supplementary Figure S2 Comparison of QQ plots to select model that fits wheat powdery mildew data from three greenhouse experiments (2010FA, 2011SA, and 2013SS) and one field experiment (2013_Field).

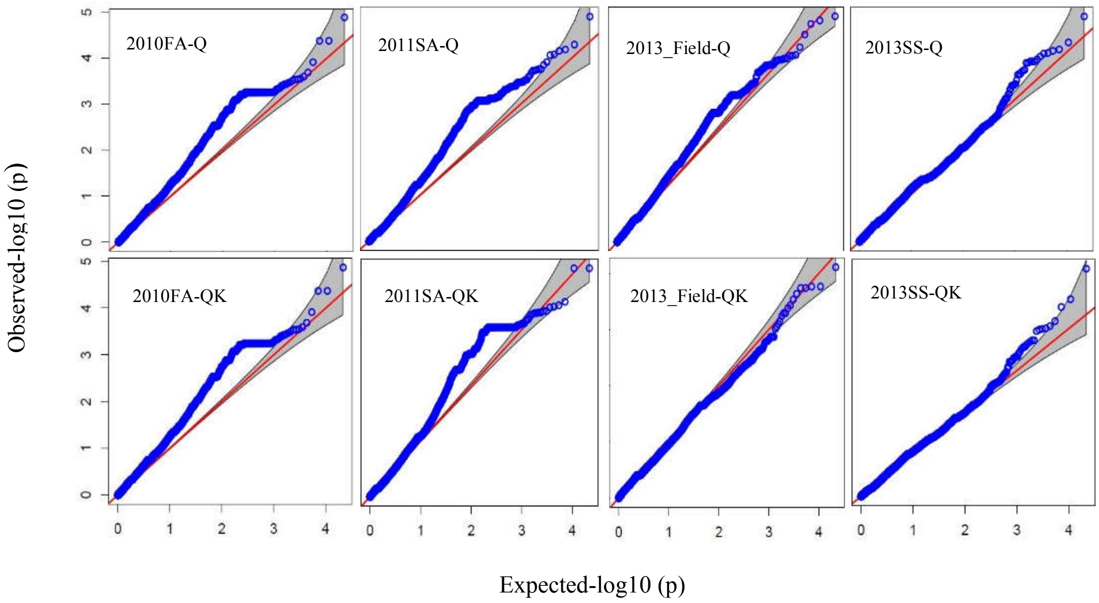

Supplementary Table S2. Detailed information for the significant markers linked to QTL for powdery mildew resistance detected from association mapping.

No.	Marker name	Chromosome	Marker Type	Position* (cM)	No.	Marker name	Chromosome	Marker Type	Position* (cM)
1	Kukri_c46010_146	1A	90K-SNP	35.41	21	RAC875_c16962_288	6A	90K-SNP	25.15
2	wsnp_Ex_c31983_40709607	1A	90K-SNP	35.41	22	Excalibur_c23748_1050	6A	90K-SNP	--
3	Ra_c5696_1556	1A	90K-SNP	44.51	23	BobWhite_c19725_1329	3B	90K-SNP	56.89
4	wsnp_Ex_c12101_19360213	1A	90K-SNP	44.51	24	wsnp_JD_c19725_17732526	3B	90K-SNP	56.89
5	BobWhite_c34661_208	1A	90K-SNP	44.51	25	Excalibur_c5298_171	3B	90K-SNP	56.89
6	wsnp_Ex_c31525_40302747	1A	90K-SNP	44.51	26	Excalibur_c5416_846	22	90K-SNP	--
7	Kukri_c4099_321	1A	90K-SNP	--	27	Ku_c71049_1180	22	90K-SNP	--
8	Jagger_c2047_362	2A	90K-SNP	59.23	28	wsnp_Ex_c13496_21243167	5B	90K-SNP	38.77
9	Ku_c57425_413	2A	90K-SNP	59.23	29	Kukri_c18702_132	5B	90K-SNP	38.77
10	CAP8_c4813_252	2A	90K-SNP	--	30	wsnp_Ex_c20988_30107609	5B	90K-SNP	38.77
11	Kukri_rep_c73477_888	2A	90K-SNP	59.23	31	BobWhite_c14258_434	1B	90K-SNP	--
12	wsnp_Ex_c2887_5330426	2A	90K-SNP	59.23	32	BS00093945_51	1B	90K-SNP	44.44
13	wsnp_Ex_c2887_5330787	2A	90K-SNP	59.23	33	JD_c12243_360	1B	90K-SNP	--
14	BS00068178_51	5A	90K-SNP	14.78	34	BobWhite_c26130_475	1B	90K-SNP	--
15	Kukri_c14889_116	5A	90K-SNP	14.78	35	BS00077837_51	1D	90K-SNP	44.44
16	GENE-3189_377	5A	90K-SNP	14.78	36	IACX6586	1D	90K-SNP	44.44
17	BS00098207_51	5A	90K-SNP	14.78	37	Kukri_c3181_2340	1D	90K-SNP	--
18	wsnp_Ex_c11621_18716254	6A	90K-SNP	25.15	38	Xscm0009	1A	SSR	--
19	Tdurum_contig9612_80	6A	90K-SNP	--	39	Xcfd95	6D	SSR	52.35
20	RFL_Contig5693_646	6A	90K-SNP	--	40	Xcfd9-2	3D	SSR	--

*The chromosome locations were assigned based on W7984 reference sequence map.
